# A novel multiple kernel fuzzy topic modeling technique for biomedical data

**DOI:** 10.1186/s12859-022-04780-1

**Published:** 2022-07-12

**Authors:** Junaid Rashid, Jungeun Kim, Amir Hussain, Usman Naseem, Sapna Juneja

**Affiliations:** 1 Department of Computer Science and Engineering, Kongju National University, Cheonan, 31080 Korea; 2 Department of Software, Department of Computer Science and Engineering, Kongju National University, Cheonan, 31080 Korea; 3 Data Science and Cyber Analytics Research Group, Edinburgh Napier University, Edinburgh, EH11 4DY UK; 4 School of Computer Science, University of Sydney, Sydney, Australia; 5 Department of Computer Science, KIET Group of Institutions, Dehli NCR, Ghaziabad, India

**Keywords:** Topic modeling, Medical data, Multiple kernel fuzzy topic modeling, MKFTM, Classification, Clustering

## Abstract

**Background:**

Text mining in the biomedical field has received much attention and regarded as the important research area since a lot of biomedical data is in text format. Topic modeling is one of the popular methods among text mining techniques used to discover hidden semantic structures, so called topics. However, discovering topics from biomedical data is a challenging task due to the sparsity, redundancy, and unstructured format.

**Methods:**

In this paper, we proposed a novel multiple kernel fuzzy topic modeling (MKFTM) technique using fusion probabilistic inverse document frequency and multiple kernel fuzzy c-means clustering algorithm for biomedical text mining. In detail, the proposed fusion probabilistic inverse document frequency method is used to estimate the weights of global terms while MKFTM generates frequencies of local and global terms with bag-of-words. In addition, the principal component analysis is applied to eliminate higher-order negative effects for term weights.

**Results:**

Extensive experiments are conducted on six biomedical datasets. MKFTM achieved the highest classification accuracy 99.04%, 99.62%, 99.69%, 99.61% in the Muchmore Springer dataset and 94.10%, 89.45%, 92.91%, 90.35% in the Ohsumed dataset. The CH index value of MKFTM is higher, which shows that its clustering performance is better than state-of-the-art topic models.

**Conclusion:**

We have confirmed from results that proposed MKFTM approach is very efficient to handles to sparsity and redundancy problem in biomedical text documents. MKFTM discovers semantically relevant topics with high accuracy for biomedical documents. Its gives better results for classification and clustering in biomedical documents. MKFTM is a new approach to topic modeling, which has the flexibility to work with a variety of clustering methods.

## Background

Many medical records are mostly in text format, and these documents must be analyzed to find meaningful information. According to the National Science Foundation, managing and analyzing scientific data on a large scale is a major challenge for data and future research [1]. The massive amount of biomedical text data can be a valuable source of knowledge for biomedical researchers. Biomedical texts contain unstructured information, such as scientific publications and brief case reports. Text mining seeks to discover knowledge from unstructured text sources by utilizing tools and techniques from a variety of fields such as machine learning, information extraction, and cognitive science. Text mining is a promising approach and great scientific interest in the biomedical domain. These text documents in biomedical require new tools to search for related documents in a collection of documents. Today's biomedical text data is created and stored very quickly. Such as, in 2015, the number of papers available on the PubMed website exceeded six million. The average record of hospital discharges in the United States is more than 30 million [2]. Therefore, companies can save annual costs by using advanced data analysis technology based on machine learning for biomedical text data. Therefore, there is a need to produce efficient topic modeling techniques through advanced machine learning to discover hidden topics in complex biomedical texts.

One way to represent biomedical text documents in natural language processing is called the bag-of-words (BOW) model. The BOW model corresponds to the frequency of words reflected in the matrix of a document collection, and word order in the document does not affect the BOW model. If the document has a vocabulary much shorter than a matrix, it is called a sparse matrix [3].

In text mining, all text corpora are processed, not just biomedical ones. There are several text mining applications such as Medline and PubMed. However, because most biomedical data is in unstructured text format, analyzing that unstructured data is a difficult task. Numerous text mining techniques are developed for the biomedical data domain that processes unstructured data into structured data. In the unstructured existence of biomedical text data, topic modeling techniques such as latent Dirichlet allocation (LDA) [4], Latent semantic analysis(LSA) [5], Fuzzy latent semantic analysis (FLSA) [6] and Fuzzy k-means topic model (FKTM)[7] are developed to analyze biomedical text data. LDA performs better in the classification of clinical reports [8]. LDA is used in a various applications, including the classification of genome sequence [9], the discovery of discussion concepts in social networks [10], patient data modeling [11], topic extraction from medical reports [12], the discovery of scientific data and biomedical relationships [13, 14]. The LDA method finds important clinical problems and formats clinical text reports in another investigation [15]. In other work, [16] used topic modeling to express scientific reports efficiently, allowing the analysis of the collections more quickly. Probabilistic-based topic modeling is applied to find the basic topics of the biomedical text collection. Topic process models are utilized in a variety of activities such as computer linguistics, overview for source code documents [17], product review brief opinion [18], description of a thematic revolution [19], discovery aspects of document analysis [20], sentiment analysis [21] and Twitter text message analysis [22]. LSA discovers clinical records from psychiatric narratives [19]. Semantic space is developed from psychological terms. LSA is also used to reveal semantic insights and ontology domains that are used to build a speech act model for spoken speech [23]. LSA also excels at topic identification and segmentation in clinical studies [24]. The RedLDA topic model is used in the biomedical field to determine redundancy in patient information data [25]. The latent semantic analysis (LSA) is an automatic analysis of the summary of clinical cases [26]. Topic models are used in biomedical data for a variety of purposes, such as finding hidden theme in documents and searching documents [27], document classification [28], and document analysis [29]. Topic modeling is an effective way to extract biomedical text, but word redundancy negatively affects topic modeling [30], and since most biomedical documents are duplicate words, it still needs improvement [31]. Answering biological factoid questions is a crucial part of the biomedical question answering domain [32]. In [33] relationship are discover from the text data.

Clustering is a process utilized in the biomedical investigation to extract meaningful information from large datasets. Fuzzy clustering is another way for hard clustering algorithms to divide data into subgroups with similar aspects [34]. The nonlinear nature of fuzzy clusters and the flexibility of large-scale data clusters distinguish them from hard clustering. It offers more accurate solutions for partitioning and additional options for decision-making. Fuzzy clustering is a type of computation based on fuzzy logic, reflecting the probability or score of a data item belonging to multiple groups. Once the data is partitioned, the centers of the clusters are moved instead of the data points. Clustering is commonly done in order to identify patterns in large datasets and to retrieve valuable information [35]. Fuzzy grouping techniques are frequently used in a variety of applications where grouping of overlapping and ambiguous elements is required. In the biomedical field, some experience has been gathered in diagnosis and decision support systems, where a wide range of measurements is used as the data entry space, and a decision result is formed by suitably grouping the data symptom. Fuzzy clustering is a technique used for various applications such as medical diagnosis, biomedical signal classification, and diabetic neuropathy [36, 37]. It can also detect topics from biomedical documents and make informed decisions about radiation therapy. Fuzzy clustering has several uses in the biomedical field, especially in image processing and pattern recognition, but it is rarely used in topic modeling. In this study, we presented a multiple kernel fuzzy topic modeling method for biomedical text data. The main contributions made to this research are summarized below.

- We proposed a novel multiple kernel fuzzy topic modeling (MKFTM) technique, which solves the problem of sparsity and redundancy in biomedical text mining.
- We proposed a FP-IDF (fusion probabilistic inverse document frequency) for global term weights, which is very effective for filtering out common high frequency words.
- We conduct extensive experiments and show that MKFTM achieves better classification and clustering performance than latest state-of-the-art topic models including LDA, LSA, FLSA, and FKTM.
- We also compare the execution time of MKFTM and shows that its execution time is stable for different topics.

## Materials and method

We described our proposed multiple kernel fuzzy topic modeling method that discover the uncover hidden topics in biomedical text documents. The two main approaches to clustering are hard clustering and fuzzy (soft) clustering. In clustering, objects are divided, and each object is a partition. MKFTM handles multi-kernel fuzzy view, a unique method for topic modeling, and validates over various experiment for medical documents. LDA performance is better for topic modeling, but redundancy always negatively impacts its performance. Therefore, MKFTM has the potential to deal with redundancy issues and discover more accurate topics in biomedical documents with higher performance than competitors like LDA and LSA.

### Multiple kernel fuzzy topic modeling (MKFTM)

The documents and words in these document are fuzzy groups in multiple clusters. Fuzzy logic is an extension of the classic 1 and 0 logic to a truth value between 1 and 0. Through MKFTM, documents and words are fuzzily clustered, with each cluster being a topic. The documents are multi-distribution across topics, and clusters are the topics in these documents. MKFTM finds the different matrices of probability. The proposed MKFTM are the following steps:

### Pre-processing

This step performs a preprocessing of the document input text collection. There is a lot of noise in text documents, such as word transforms, word shape transforms, special characters, punctuation marks, and stop words that add noise. Several pre-processing steps are used to clean up the text data. The punctuation is removed from the document collection. Text data is converted to lowercase and documents are tokenized. After that, short, empty words with fewer characters are removed. Also, the words are normalized through the Porter Stemmer [38].

### Bag-of-words (BOW) and term weighting

The bag-of-words model represents text documents and extracts features from text documents for machine learning algorithms. BOW is a systematic method for calculating document words count [39]. After collecting and preprocessing the document's text, the BOW model is applied. BOW model converts unstructured text data into word-based structured data, ignoring the grammar in information retrieval [40]. The ${\varvec{m}}$ documents contain the word ${\varvec{k}}$ finding the association between words and document. Also, the frequency of ${\varvec{k}}$ words in documents ${\varvec{m}}$ is calculated. Equation 1 represent the words ${\varvec{k}}$ frequencies in documents ${\varvec{m}}$. The ${\varvec{k}}^{{\varvec{n}}}$ means the words ${\varvec{k}}$ count in ${\varvec{n}}$ documents. The ${\varvec{n}}_{{\varvec{i,j}}}$ means the count of words in matrix ${\varvec{i}},{\varvec{j}}$. The ${\varvec{k}}_{{\varvec{j}}}$ means numbers that the numbers of words count in rows. The ${\varvec{tf}}$ is term frequency.

1 $$tf_{{i,j}} = \mathop \sum \limits_{{k^{n} }}^{{n_{{i,j}} }} k,j$$

Local terms are weighted after applying BOW and the term frequency method is another local term method. The term frequency [41] evaluate evaluates the frequency with which the term appears in a document. Because each document is of different lengths, more terms may appear in longer documents than shorter ones. Equation 2 shows a typical weighting term that uses a vector field of normalization coefficients. The term weight, which reduces these terms, is essential and assigned $w_{dk}$ that constantly varies from 0 to 1. Here, $d$ represents a document, k defines the term and $w_{dk}$ means k terms of d documents in words w. Weight is used in the most important terms and zero is used in the least important terms. In some cases, the use of a standard weight assignment may be useful, and the weighting term depends on many impacts on the weights, using different terms individually within each vector.

2 $$\frac{{w_{dk} }}{{\sqrt {\mathop \sum \nolimits_{vector} (w_{d} i)^{2} } }}$$

This shows the weight $w$ of the $k$ term. If a term index $k_{i}$ frequency $f_{i,j}$ appear in the document $d_{j}$, the general frequency $F_{i}$ of the k terms is well-defined in Eq. 3.

3 $${\text{F}}_{{\text{i}}} = \mathop \sum \limits_{{\left( {{\text{j}} = 1} \right)}}^{{\text{N}}} {\text{f}}_{{{\text{i}},{\text{j}}}}$$

$N$ is a numbers of document in a large set of text corpus. The frequency of document term kiki refers to the number of $n_{i}$ documents occurrence and ni < Fi.

### Fusion probabilistic inverse document frequency (FP-IDF)

The weight of global term (GTW) is estimated at this stage. GTW provide "discrimination values" for all terms. The less frequent terms in document collection are more discriminating [42]. The $tf_{ij}$ symbol determine the number of time word $i$ appears in document j. The number of documents is indicated by $N$ and $n_{i}$ is total number of documents appearing in the $i$ term. GTW is calculated by finding the b(tfij) and Pij using Eq. 4, 5.

4 $$b\left( {tf_{ij} } \right) = \left\{ {\begin{array}{*{20}l} 1 \hfill & {if} \hfill & {tf_{ij} > 0} \hfill \\ 0 \hfill & {if} \hfill & {tf_{ij} = 0} \hfill \\ \end{array} } \right\}$$

5 $$P_{ij} = \frac{{tf_{ij} }}{{\mathop \sum \nolimits_{j} tf_{ij} }}$$

The b(tfij) and Pij are used to calculate the fusion probabilistic inverse document frequency. We proposed a FP-IDF by combining the hybrid inverse documents frequency $\left( {{\text{Hybrid}} - {\text{IDF}}} \right)$ and probabilistic Inverse documents frequency $\left( {{\text{Probablistic}} - {\text{IDF}}_{ } } \right)$ for weighting global term. Equations 6 and 7 show the formula for ${\text{Hybrid}} - {\text{IDF}}$ and ${\text{Probablistic}} - {\text{IDF}}_{ }$.

6 $$Hybrid - IDF = \log \left( {\max_{{\left\{ {t^{{\prime }} \in d} \right\}n_{{t^{{\prime }} }} }} \left( {\frac{N}{{n_{t} }}} \right)} \right)$$

7 $$Probablistic - IDF = \log \left( {\frac{{N - n_{t} }}{{n_{t} }}} \right)$$

8 $$Fusion\,Probablistic - IDF = \log \left( {\max_{{\left\{ {t^{{\prime }} \in d} \right\}n_{{t^{{\prime }} }} }} \left( {\frac{N}{{n_{t} }}} \right)} \right) + \log \left( {\frac{{N - n_{t} }}{{n_{t} }}} \right)$$

Use the product property of logarithms, ${\text{log}}_{{\text{b}}} {\text{x}} + {\text{log}}_{{\mathrm{b}}} {\text{y}} = {\text{log}}_{{\mathrm{b}}} {\text{xy}}$.

9 $$Fusion\,Probablistic - IDF = \log \left( {\max_{{\left\{ {t^{{\prime }} \in d} \right\}n_{{t^{{\prime }} }} }} \left( {\frac{N}{{n_{t} }}\frac{{N - n_{t} }}{{n_{t} }}} \right)} \right)$$

Combine $$\max _{{\left\{ {{\text{t}}^{{\prime }} \in {\text{d}}} \right\}{\text{n}}_{{{\text{t}}^{{\prime }} }} }}$$ and $$\frac{{\text{N}}}{{{\mathrm{n}}_{{\text{t}}} }}$$

10 $$Fusion\,Probablistic - IDF = \log \left( {\frac{{\max_{{\left\{ {t^{{\prime }} \in d} \right\}n_{{t^{{\prime }} }} }} N}}{{n_{t} }} \cdot \frac{{N - n_{t} }}{{n_{t} }}} \right)$$

Multiply $$\frac{{\max _{{\left\{ {{\text{t}}^{{\prime }} \in {\text{d}}} \right\}{\text{n}}_{{{\text{t}}^{{\prime }} }} }} {\text{N}}}}{{{\mathrm{n}}_{{\text{t}}} }}\,\,{\text{and}}\,\,\frac{{{\text{N}} - {\text{n}}_{{\text{t}}} }}{{{\mathrm{n}}_{{\text{t}}} }}$$

11 $$Fusion\,Probablistic - IDF = \log \left( {\frac{{\max_{{\left\{ {t^{{\prime }} \in d} \right\}n_{{t^{{\prime }} }} }} N\left( {N - n_{t} } \right)}}{{n_{t} n_{t} }}} \right)$$

Raise ${\text{n}}_{{\text{t}}}$ to the power of 1.

12 $$Fusion\,Probablistic - IDF = \log \left( {\frac{{\max_{{\left\{ {t^{{\prime }} \in d} \right\}n_{{t^{{\prime }} }} }} N\left( {N - n_{t} } \right)}}{{n_{t}^{1} n_{t} }}} \right)$$

Raise ${\text{n}}_{{\text{t}}}$ to the power of 1.

13 $$Fusion\,Probablistic - IDF = \log \left( {\frac{{\max_{{\left\{ {t^{{\prime }} \in d} \right\}n_{{t^{{\prime }} }} }} N\left( {N - n_{t} } \right)}}{{n_{t}^{1} n_{t}^{1} }}} \right)$$

Use the power rule ${\text{a}}^{{\text{m}}} {\text{a}}^{{\text{n}}} = {\text{a}}^{{{\text{m}} + {\text{n}}}}$ to combine exponents.

14 $$Fusion\,Probablistic - IDF = \log \left( {\frac{{\max_{{\left\{ {t^{{\prime }} \in d} \right\}n_{{t^{{\prime }} }} }} N\left( {N - n_{t} } \right)}}{{n_{t}^{1 + 1} }}} \right)$$

Add 1 and 1. We proposed a FP-IDF in Eq. 15.

15 $$FP - IDF = \log \left( {\frac{{\max_{{\left\{ {t^{{\prime }} \in d} \right\}n_{{t^{{\prime }} }} }} N\left( {N - n_{t} } \right)}}{{n_{t}^{2} }}} \right)$$

### Principal component analysis (PCA)

After the FP-IDF global terms weighting method, the PCA is used. The PCA [43] technique has been used to avoid large-scale adverse effects in the weighting of global terms. This method removes redundant dimensions from the data and retains only the most important data dimensions. The PCA calculates the new variable that refers to the principal component, resulting from the integrated integration of the initial variables.

### Multiple Kernel fuzzy C-means clustering

At this step, the multiple kernel fuzzy c-means clustering algorithm [44] is used for fuzzily group documents, which is represented by GTW method. In multiple kernel fuzzy c-means clustering algorithm B is a data point,${\text{Y}} = \left\{ {{\text{Y}}_{{\text{i}}} } \right\}_{{{\text{i}} = 1}}^{{\text{B}}}$, kernel function $\left\{ {{\text{G}}_{{\text{g}}} } \right\}_{{{\text{g}} = 1}}^{{\text{S}}}$, numbers of desired clusters are F and output membership matrix ${\text{V}} = \left\{ {{\text{v}}_{{{\text{if}}}} } \right\}_{{{\text{i}},{\text{f}} = 1}}^{{{\text{B}},{\text{F}}}}$ with weight $\left\{ {{\text{Z}}_{{\text{g}}} } \right\}_{{{\text{g}} = 1}}^{{\text{S}}}$ for kernels. The multiple kernel fuzzy c-means have the following steps:

1. Procedure multiple kernel fuzzy c-means MKFCM (Data Y, Clusters F, Kernels $\left\{ {Z_{g} } \right\}_{g = 1}^{S}$)
2. Membership matrix initialization $V^{\left( 0 \right)}$.
3. Repeat
4. $$\overset{\lower0.5em\hbox{$\smash{\scriptscriptstyle\frown}$}}{v}_{if}^{l} = \frac{{u_{ic}^{{(l)^{s} }} }}{{\mathop \sum \nolimits_{i = 1}^{B} v_{if}^{{(l)^{s} }} }}$$, $$\triangleright$$ Calculate the normalized membership.
5. $\triangleright$ Calculate Coefficients Eq. 16
  $${\upalpha }_{{{\text{ifg}}}} = {\text{G}}_{{\text{g}}} \left( {{\text{y}}_{{\text{i}}} ,{\text{y}}_{{\text{i}}} } \right) - 2\mathop \sum \limits_{{{\text{J}} = 1}}^{{\text{B}}} \overset{\lower0.5em\hbox{$\smash{\scriptscriptstyle\frown}$}}{\text{v}}_{{{\text{jf}}}} {\text{G}}_{{\text{g}}} \left( {{\text{y}}_{{\text{i}}} ,{\text{y}}_{{\text{j}}} } \right) + \mathop \sum \limits_{{{\text{j}} = 1}}^{{\text{B}}} \mathop \sum \limits_{{{\text{j}}^{\prime} = 1}}^{{\text{B}}} \overset{\lower0.5em\hbox{$\smash{\scriptscriptstyle\frown}$}}{\text{v}}_{{{\text{jf}}}} \overset{\lower0.5em\hbox{$\smash{\scriptscriptstyle\frown}$}}{\text{v}}_{{{\text{j}}^{{\prime }} {\text{f}}}} {\text{G}}_{{\text{g}}} \left( {{\text{y}}_{{\text{j}}} ,{\text{y}}_{{{\text{j}}^{{\prime }} }} } \right)\quad (16)$$
6. for (i = 1…B; f = 1..F; g = 1..S) do
7. $$\alpha_{ifg} \mathop \leftarrow \limits^{\quad \quad } G_{g} (y_{i} ,y_{i} ) - 2\mathop \sum \limits_{J = 1}^{B} \overset{\lower0.5em\hbox{$\smash{\scriptscriptstyle\frown}$}}{v}_{jc} G_{g} \left( {y_{i} ,y_{j} } \right) + \mathop \sum \limits_{j = 1}^{B} \mathop \sum \limits_{{j^{\prime} = 1}}^{B} \overset{\lower0.5em\hbox{$\smash{\scriptscriptstyle\frown}$}}{v}_{jf} \overset{\lower0.5em\hbox{$\smash{\scriptscriptstyle\frown}$}}{v}_{{j^{{\prime }} f}} G_{g} \left( {y_{j} ,y_{{j^{{\prime }} }} } \right)\quad (17)$$
8. end for
9. $\triangleright$ Calculate coefficient by Eq. 18.
10. for (g = 1…S) do
11. $$\beta_{k} \leftarrow \mathop \sum \limits_{i = 1}^{B} \mathop \sum \limits_{f = 1}^{F} \left( {v_{if}^{(l)} } \right)^{s} \alpha_{ifg} \quad (18)$$
12. end for
13. $\triangleright$ Update weights by Eq. 19.
14. for (g = 1…S) do
15. $$z_{g}^{(l)} \leftarrow \frac{{\frac{1}{{\beta_{g} }}}}{{\frac{1}{{\beta_{1} }} + \frac{1}{{\beta_{2} }} \cdots \frac{1}{{\beta_{S} }}}}\quad (19)$$
  end for
16. $\triangleright$ distance calculate by Eq. 20.
17. for (i = 1…B;c = 1..F) do
18. $$T_{if}^{2} \leftarrow \mathop \sum \limits_{g = 1}^{S} \alpha_{ifg} \left( {z_{g}^{\left( l \right)} } \right)^{2} \quad (20)$$
19. end for
20. $\triangleright$ update memberships Eq. 21
21. for (i = 1…B;f = 1..F) do
22. $$v_{if}^{(l)} \leftarrow \frac{1}{{\mathop \sum \nolimits_{{f^{\prime} = 1}}^{F} \left( {\frac{{D_{if}^{2} }}{{D_{{if^{\prime}}}^{2} }}} \right)^{{\frac{1}{s - 1}}} }}\quad (21)$$
23. end for
24. until $$\left| {\left| {Vl - V^{l - 1} } \right|} \right|$$< ∋ 
25. return $$V^{(l)} ,\left\{ {z_{g}^{\left( l \right)} } \right\}_{g = 1}^{S}$$.
26. end procedure

### Probabilistic distribution of documents

The document term matrix, along with the GTW method (matrix of words × documents), find the probability of a document P(Dj), calculated by Eq. 22. Here $i$ represents the various documents.

22 $$P(D_{j} ) = \frac{{\sum\nolimits_{i = 1}^{m} {(W_{i} ,D_{j} )} }}{{\sum\nolimits_{i = 1}^{m} {\sum\nolimits_{j = 1}^{n} {(W_{i} ,D_{j} )} } }}$$

### Probabilistic distribution of the topics for documents

The probabilities of obtaining the $j$ documents in the $k$ topic are $P(D_{j} |T_{k} )$ through P(Tk|Dj) with P(Dj), as described in Eq. 23.

23 $$P(D_{j} ,T_{k} ) = P(T_{k} |D_{j} ) \times P(D_{j} )$$

Since, finding the ${ }P(D_{j} |T_{k} )$, normalized the P(D,T) for each topic through Eq. 24.

24 $$P(D_{j} |T_{k} ) = \frac{{P(D_{j} ,T_{k} )}}{{\sum\nolimits_{j = 1}^{n} {P(D_{j} ,T_{k} )} }}$$

### Probabilistic distribution of words in documents

This step calculates the probability of a word $i$ in the $j$ document applying Eq. 25.

25 $$P(W_{i} |D_{j} ) = \frac{{P(W_{i} ,D_{j} )}}{{\sum\nolimits_{i = 1}^{m} {P(W_{i} ,D_{j} )} }}$$

### Probabilistic distribution of words in topics

The probabilities of word $i$ in topic $k$ $P(W_{i} |T_{k} )$ through $P(D_{j} |T_{k} )$ and P(Wi|Dj) is calculated through Eq. 26.

26 $$P(W_{i} |T_{k} ) = \mathop \sum \limits_{j = 1}^{n} P(W_{i} ,D_{j} ) \times P(D_{j} |T_{k} )$$

### Datasets

In this research, we used six state-of-the-art datasets, which are publicly available. The first dataset is a medical abstract of the English scientific corpus from MuchMore Springer Bilingual Corpus,^1^ a labeled dataset. We used two categories of journals, including the federal health standard and arthroskopie, for experimentation. Table 1 shows the statistics of datasets.

- The medical abstract from MeSH categories from Ohsumed Collection^2^ is a second labeled corpus dataset. The experiments are conducted in three categories: virus disease, bacterial infection, and mycoses.
- Biotext [45] is the third dataset, containing summaries of diseases and treatments collected from Medline.
- The fourth data set is GENIA corpora [46], abstracts collection from Medline papers describing the molecular biology literature.
- The fifth is the redundant corpus of synthetic WSJ and is generally used in natural language processing (NLP) [47, 48].
- The six datasets are health news tweets^3^ (T-datasets), an unlabeled dataset.

**Table 1:** Datasets statistics

Datasets	Documents (Preprocess)	Words	Unique words
MuchMore Springer	1527	19,835	5008
Ohsumed	2092	22,669	13,238
Genia	2000	21,560	17,834
Biotext	40	25,921	10,267
Twitter	58,927	395,636	25,309
WSJ	1300	680 K	36 K

## Results

### Experimental performed

We performed the classification, clustering, execution time and redundancy issues for experiments. We used six state-of-the-art datasets for experiments. The first two datasets Muchmore springer bilingual and Ohsumed Collection, are labeled datasets. Therefore, it's used for classification. The other two datasets Biotext and Genia are unlabeled. Hence, it's used for clustering. The redundant corpus of synthetic WSJ is used for the redundancy issue comparison because in literature this dataset is mostly consider for redundancy issue. Therefore, we used the same dataset for fair comparison. The execution time is compared to the health news tweets dataset, containing more documents.

### Experimental setup

We used the laptop core i7 computer with 16 GB RAM and MATLAB software for experiments.

### Baseline topic models

In this section, our proposed MKFTM topic model is compared with the state-of-the-art LDA [4], LSA [5], FLSA [6] and FKTM [7] topic models. Experiments are performed for both classification and clustering. We also compare our proposed topic model with RedLDA [25] and FKTM, which are used for redundancy problems.

### Classification of documents

The first classification evaluation is performed with Bayesian optimization for two datasets, including MuchMore Springer Bilingual Corpus and Ohsumed Collection. Optimization refers to searching for points to minimize functions with real value, known as objective functions. The bayes optimization is a gauss-process objective function model that evaluates the objective functions. Bayesian optimization minimizes cross-validation error. MATLAB fit function is used for Bayesian optimization. MKFTM performance is compared to LDA, LSA, FLSA, and Fuzzy k-means topic models using a tenfold cross-validation method. Document classification is performed on topic probabilities for document P(T|D) through discriminant analysis machine learning classifier [49] using Bayes optimization. Discriminant analysis is described in Eq. 27

27 $${\mathbf{y}} = {\mathbf{arg}} {\mathbf{min}}_{{{\mathbf{y}} = 1, \ldots .{\mathbf{K}}}} \mathop \sum \limits_{{\mathbf{k}}}^{{\mathbf{K}}} {\mathbf{p}}\left( {{\mathbf{k}}{|}{\mathbf{x}}} \right){\mathbf{C}}({\mathbf{y}}|{\mathbf{k}})$$

The $$\user2{\overset{\lower0.5em\hbox{$\smash{\scriptscriptstyle\frown}$}}{y} }$$ represent the expected classes and $${\varvec{k}}$$ is number of classes. The $$\user2{\overset{\lower0.5em\hbox{$\smash{\scriptscriptstyle\frown}$}}{p} }({\varvec{k}}|{\varvec{x}})$$ is the posterior probability of class $${\varvec{k}}$$ and observations x. The Cy|k is the classification cost and observation $${\varvec{y}}$$ with the true class $${\varvec{k}}$$. The discriminant analysis classifies the document features with different topics such as 50, 100, 150 and 200. MKFTM performance of classification is measured using precision, recall, accuracy, and F1-score. Precision, recall, accuracy and F1 measurements are used to verify the performance of the MKFTM. The classification results of two datasets labeled MuchMore Springer and Ohsumed are shown in Tables 2 and 3. The results of the MKFTM classification are compared with the latest LDA, LSA, FLSA and FKTM state-of-the-art topic models for the biomedical text corpora.

**Table 2:** Classification results (muchmore springer bilingual corpus)

Method	AC (%)	Precision	Recall	F1-Score	K
LSA [5]	57.65	0.6667	0.7221	0.6933	50
LDA [4]	60.95	0.6938	0.7356	0.7141	50
FKLSA(Entropy) [6]	97.66	0.955	0.9554	0.977	50
FKLSA(IDF) [6]	95.90	0.937	0.935	0.959	50
FKLSA(Normal) [6]	91.22	0.890	0.894	0.912	50
FKLSA(ProbIDF) [6]	97.66	0.954	0.953	0.977	50
FKTM [7]	98.29	0.9880	0.9883	0.9880	50
**MKFTM**	**99.04**	**0.9975**	**0.9978**	**0.9975**	**50**
LSA [5]	56.19	0.6676	0.6791	0.6733	100
LDA [4]	58.85	0.6854	0.7011	0.6932	100
FKLSA(Entropy) [6]	96.49	0.943	0.942	0.965	100
FKLSA(IDF) [6]	98.24	0.961	0.960	0.982	100
FKLSA(Normal) [6]	92.39	0.902	0.900	0.924	100
FKLSA(ProbIDF) [6]	97.66	0.955	0.952	0.977	100
FKTM [7]	98.87	0.9879	0.9841	0.9844	100
**MKFTM**	**99.62**	**0.9974**	**0.9936**	**0.9939**	**100**
LSA [5]	62.67	0.7091	0.7536	0.7285	150
LDA [4]	59.23	0.6991	0.6791	0.6890	150
FKLSA(Entropy) [6]	95.90	0.937	0.935	0.959	150
FKLSA(IDF) [6]	97.66	0.955	0.952	0.977	150
FKLSA(Normal) [6]	95.32	0.932	0.931	0.953	150
FKLSA(ProbIDF) [6]	97.07	0.950	0.952	0.971	150
FKTM [7]	98.97	0.9822	0.9882	0.9886	150
**MKFTM**	**99.69**	**0.9917**	**0.9976**	**0.9980**	**150**
LSA [5]	60.00	0.6980	0.7020	0.9886	200
LDA [4]	63.42	0.7039	0.7765	0.7000	200
FKLSA(Entropy) [6]	97.07	0.950	0.9501	0.7384	200
FKLSA(IDF) [6]	97.66	0.955	0.9553	0.971	200
FKLSA(Normal) [6]	92.39	0.901	0.902	0.977	200
FKLSA(ProbIDF) [6]	97.66	0.955	0.950	0.924	200
FKTM [7]	98.86	0.9883	0.9870	0.977	200
**MKFTM**	**99.61**	**0.9978**	**0.9966**	**0.965**	**200**

**Table 3:** Classification results (Ohsumed collection dataset)

Method	AC (%)	Precision	Recall	F1-Score	K
LSA [5]	48.36	0.4146	0.4224	0.4185	50
LDA [4]	54.10	0.4789	0.5155	0.4970	50
FKLSA(Entropy) [6]	75.21	0.720	0.722	0.746	50
FKLSA(IDF) [6]	75.90	0.722	0.723	0.746	50
FKLSA(Normal) [6]	71.25	0.6551	0.654	0.677	50
FKLSA(ProbIDF) [6]	74.87	0.715	0.714	0.735	50
FKTM [7]	92.35	0.9236	0.9006	0.9119	50
**MKFTM**	**94.10**	**0.9431**	**0.9200**	**0.9213**	50
LSA [5]	51.37	0.4430	0.4099	0.4258	100
LDA [4]	54.92	0.4873	0.4783	0.4828	100
FKLSA(Entropy) [6]	76.24	0.727	0.726	0.747	100
FKLSA(IDF) [6]	74.35	0.701	0.703	0.726	100
FKLSA(Normal) [6]	71.08	0.670	0.674	0.694	100
FKLSA(ProbIDF) [6]	74.52	0.702	0.704	0.724	100
FKTM [7]	87.70	0.8867	0.8261	0.8553	100
**MKFTM**	**89.45**	**0.9063**	**0.8457**	**0.8747**	100
LSA [5]	52.73	0.4651	0.4969	0.4805	150
LDA [4]	57.10	0.5123	0.5155	0.5139	150
FKLSA(Entropy) [6]	74.87	0.715	0.714	0.735	150
FKLSA(IDF) [6]	76.59	0.732	0.731	0.752	150
FKLSA(Normal) [6]	72.46	0.671	0.673	0.691	150
FKLSA(ProbIDF) [6]	75.04	0.715	0.712	0.735	150
FKTM [7]	90.16	0.8788	0.9006	0.8896	150
**MKFTM**	**92.91**	**0.8984**	**0.9203**	**0.9092**	150
LSA [5]	49.73	0.4303	0.4410	0.4356	200
LDA [4]	54.37	0.4819	0.4969	0.4893	200
FKLSA(Entropy) [6]	75.21	0.720	0.721	0.740	200
FKLSA(IDF) [6]	74.18	0.705	0.704	0.725	200
FKLSA(Normal) [6]	71.94	0.671	0.673	0.683	200
FKLSA(ProbIDF) [6]	74.87	0.701	0.702	0.729	200
FKTM [7]	88.25	0.8986	0.8261	0.8608	200
**MKFTM**	**90.35**	**0.9182**	**0.8460**	**0.8802**	200

### Clustering of documents

The clustering performance is measured in two datasets, Genia and Biotext. Document clustering is performed using the k-mean clustering method of P (T | D).There are two methods for clustering validation, and internal validation method is more accurate than external validation [50]. We use the internal validation method of the Calinski-Harabasz index to evaluate multiple topics and clusters. The Calinsiki-Harabasz (CH) index [51] is a widely used internal verification method. The exponent CH is the exponent relationship where cohesion is estimated at the distance from the center point as shown in Eq. 28, where k is the number of clusters and N is the total number of observations.

28 $$CH(C) = \frac{(N - K)}{{(K - 1)}}\frac{{\sum\limits_{ck} { \in C|C_{k} |d_{e} |} \,(\overline{C}_{k} ,\overline{X})}}{{\sum\limits_{ck} { \in C\sum\limits_{xi} {C_{k} d_{e} (x^{i} ,\overline{C}_{k} )} } }}\;\;$$

**Fig. 1:**
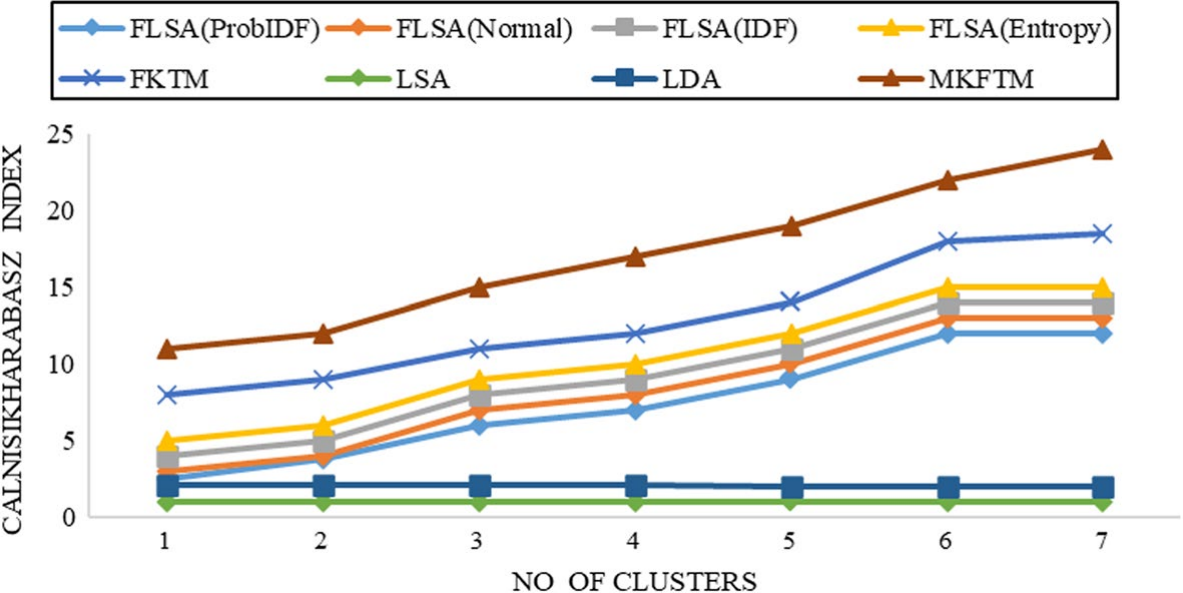
CH-index results for Genia datasets with K = 50

**Fig. 2:**
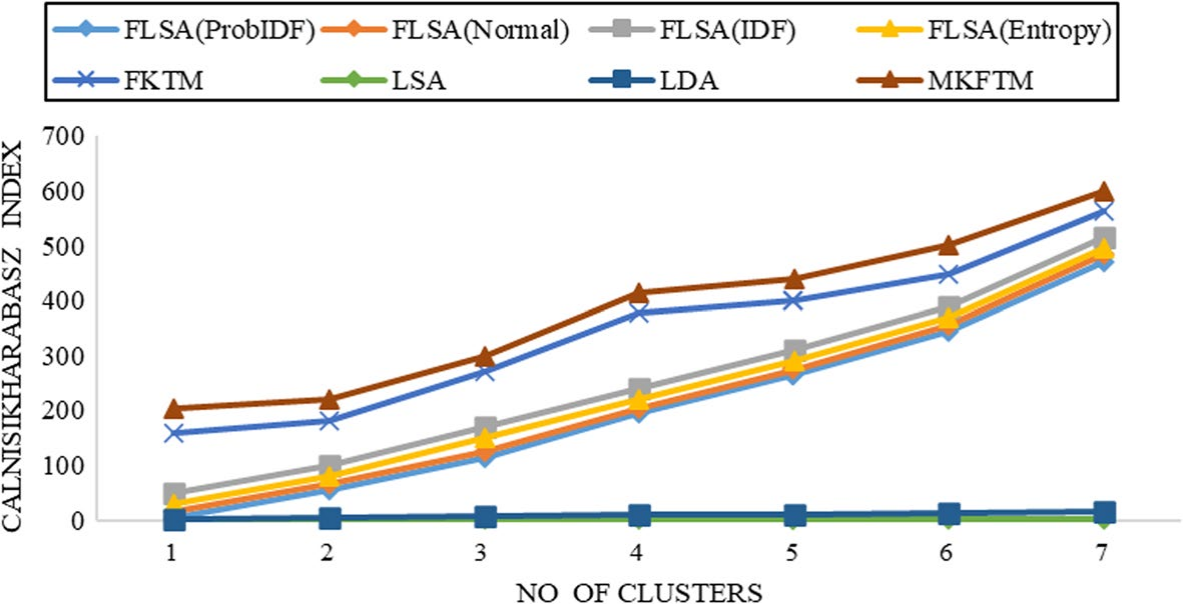
CH-index results for Genia datasets with K = 100

**Fig. 3:**
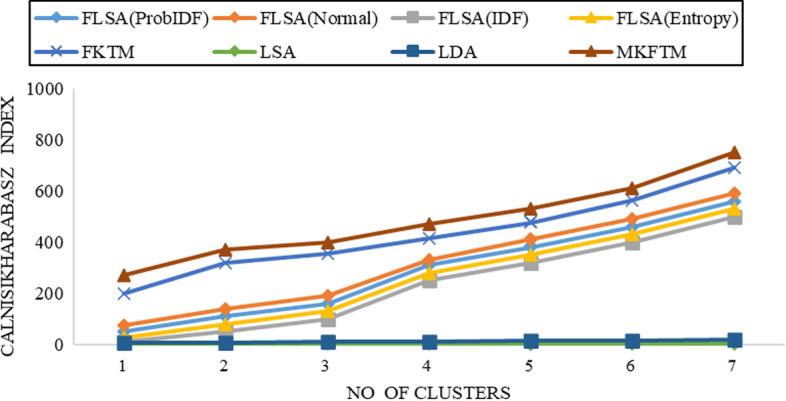
CH-index results for Genia datasets with K = 150

**Fig. 4:**
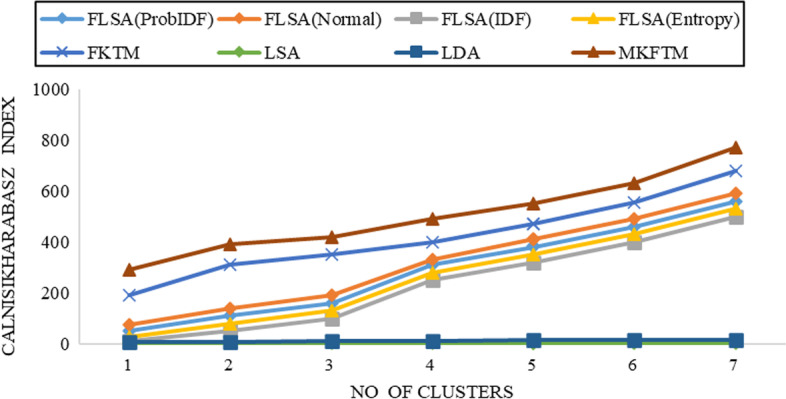
CH-index results for Genia datasets with K = 200

**Fig. 5:**
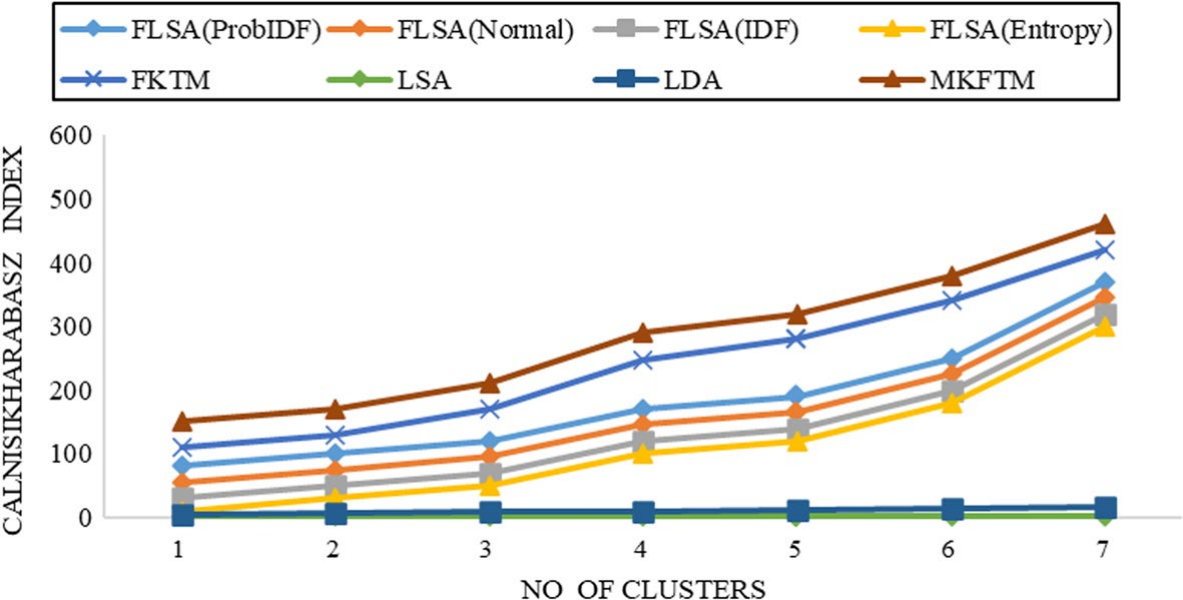
CH-index results for Biotext datasets with K = 50

**Fig. 6:**
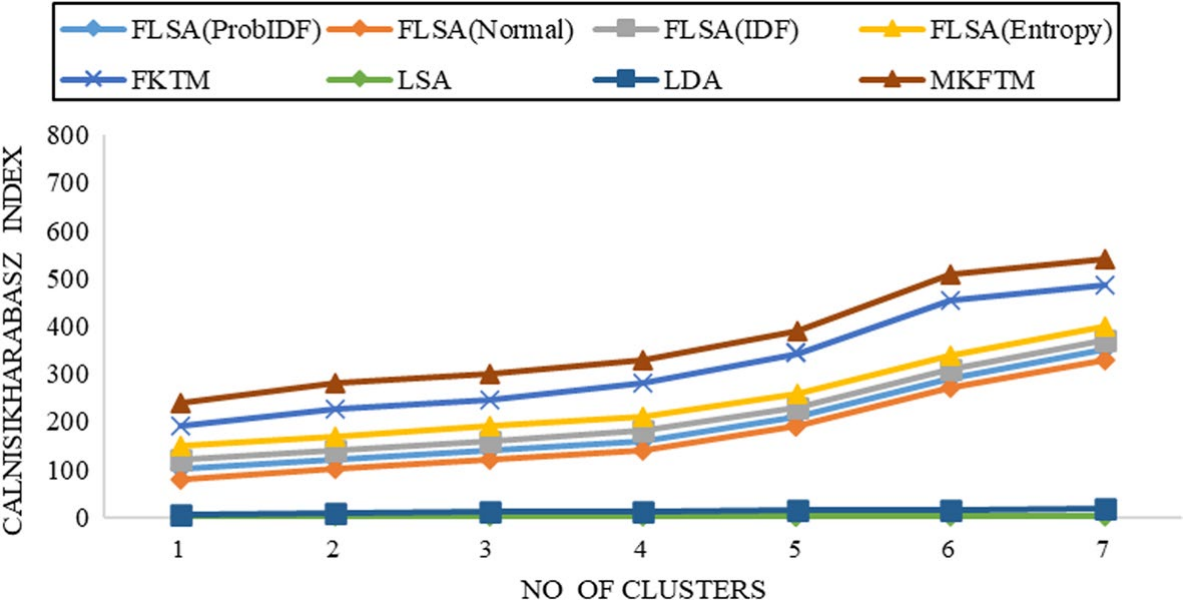
CH-index results for Biotext datasets with K = 100

**Fig. 7:**
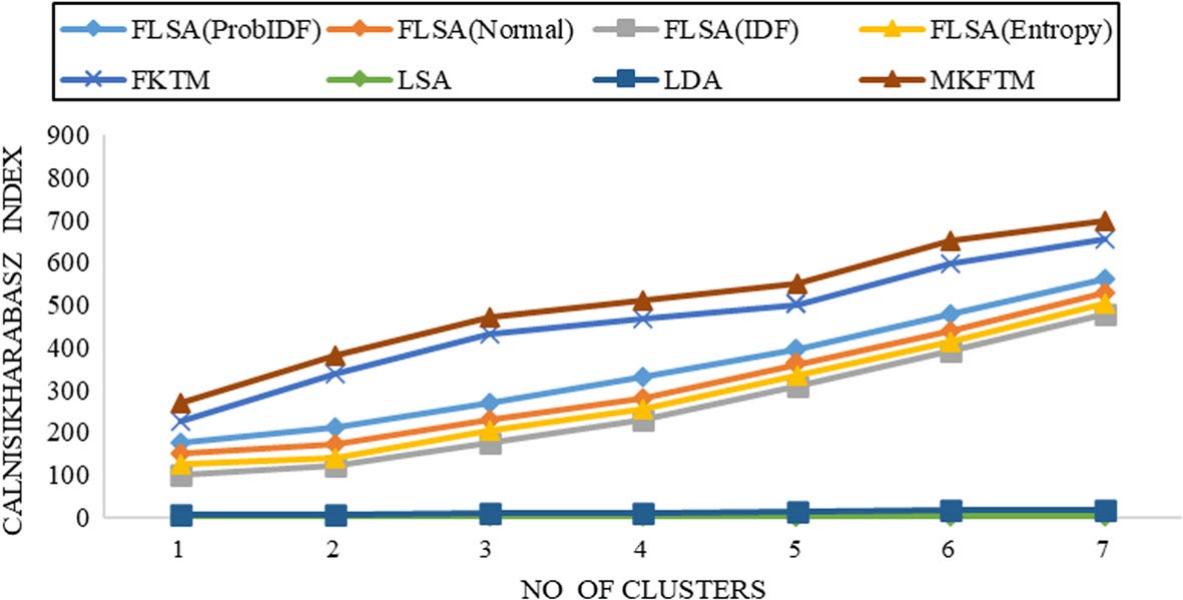
CH-index results for Biotext datasets with K = 150

**Fig. 8:**
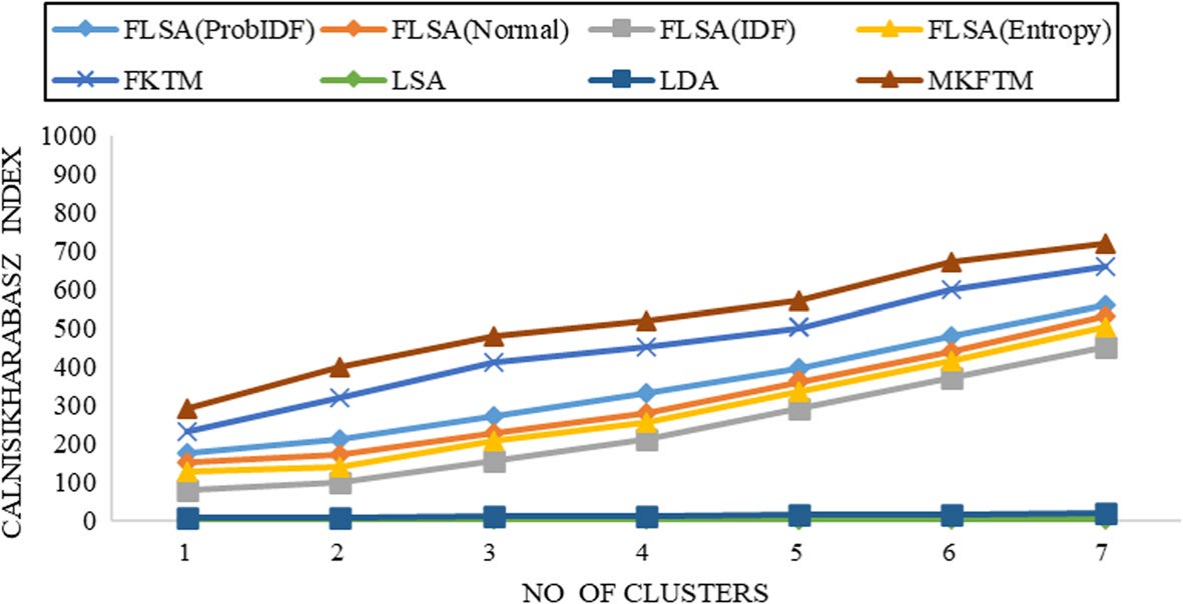
CH-index results for Biotext datasets with K = 200

The Calinsiki-Harabasz index can assess the reliability of all clusters by summing the mean square error. The highest Calinsiki-Harabasz index shows the best results of the clustering. The Calinsiki-Harabasz index gives the best results for clusters and finds the corresponding clusters that appear. Figure 1, 2, 3, 4, 5, 6, 7 and 8 shows the CH index for clustering performance in Genia and biotext datasets.

### Redundancy issue

The experiment examined the influence of the redundancy problem using a WSJ synthetic redundant corpus. MKFTM versus LDA and RedLDA developed to address redundancy issues in biomedical documents [25]. LDA, RedLDA, FKLSA, Fuzzy k-means topic model and MKFTM are trained on the same redundant WSJ synthetic corpus to compare the performance of these topic models. Table 4 shows the log-likelihood probability of WSJ dataset synthetic redundancy with topics ranging from 50 to 200.

**Table 4:** Comparison of loglikelihood for WSJ corpora

Topic Model	Log-Likelihood	No of Topics
LDA	-824,000	50
RedLDA	-810,000	50
FKTM	-789,000	50
MKFTM	-773,000	50
LDA	-814,000	100
RedLDA	-805,000	100
FKTM	-789,500	100
MKFTM	-773,600	100
LDA	-815,000	150
RedLDA	-809,000	150
FKTM	-789,200	150
MKFTM	-773,700	150
LDA	-816,000	200
RedLDA	-800,000	200
FKTM	-789,000	200
MKFTM	-773,900	200

### Execution time

**Fig. 9:**
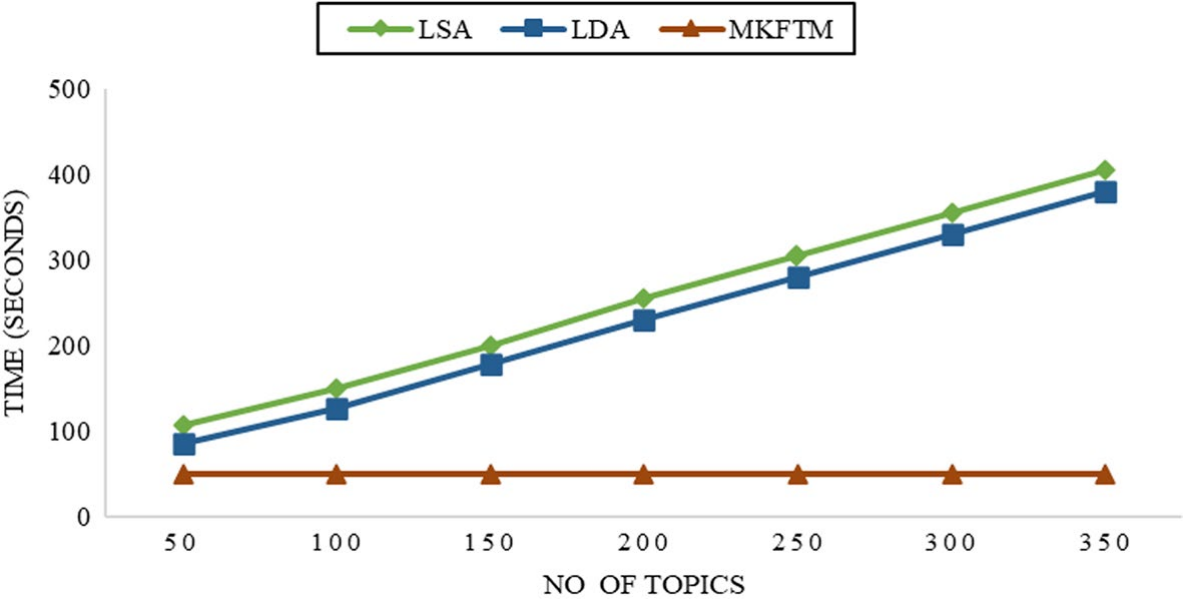
Comparison of execution times of health tweet dataset

Health News Tweets are used to compare MKFTM runtime with LDA, LSA and FLSA. Figure 9 shows the runtime performance of MKFTM, LDA and LSA.

## Discussion

The classification, clustering, redundancy issue, and execution time are used for the performance of experiments. The document classification is presented in Tables 2 and 3. Table 2 shows the classification results for the Muchmore Springer dataset. The classification results are measured with 50,100,150 and 200 numbers of topics on both datasets. MKFTM achieved 99.04%, 99.62%, 99.69%,99.61% accuracy with 50,100,150 and 200 topics on Muchmore Springer dataset. FKTM accuracy is 98.29%, 98.87%, 98.97%,98.86% with 50,100,150 and 200 topics for Muchmore Springer dataset. FLSA accuracy is higher than LDA and LSA on the Muchmore Springer dataset. FKTM accuracy is better than FLSA for the Muchmore Springer dataset. However, MKFTM achieved the highest accuracy compared to baseline topics models FKTM, FLSA, LDA, and LSA for the Muchmore Springer dataset. We also measured the precision, recall, and F1-sore score for all topic models. The precision, recall, and F1-sore score of FKTM is better than the FLSA on the Muchmore Springer dataset. LDA and LSA precision, recall, and F1-sore values are lower than FLSA for the Muchmore Springer dataset. Overall, MKFTM attained the higher scores values for precision, recall, and F1-sore for Muchmore Springer dataset. Table 3 shows the Ohsumed dataset classification results and MKFTM achieved 94.10%, 89.45%, 92.91%, 90.35% accuracy with 50,100,150, and 200 topics, respectively. For the Ohsumed dataset, FKTM accuracy is 92.35%, 87.70%, 90.16%, 88.25% for 50,100,150, and 200 topics, respectively. On the Ohsumed dataset, FLSA accuracy outperforms LDA and LSA. For the Ohsumed dataset, FKTM accuracy is highest than FLSA. However, the accuracy of MKFTM for the Ohsumed dataset is higher than the FKTM, FLSA, LDA, and LSA base topics models. In the Ohsumed dataset, FKTM outperforms FLSA in precision, recall, and F1-sore. For the Ohsumed dataset, LDA and LSA values of precision, recall, and F1-sore are lower than the FLSA. The precision, recall, and F1-sore of MKFTM is highest than the FKTM, FLSA, LDA, and LSA. The classification results show that MKFTM performance is superior to FKTM, FLSA, LDA, and LSA for Muchmore Springer and Ohsumed datasets.

Documents clustering performance is measured using the Calinski-Harabasz index for Genia and Biotext Datasets with 50,100,150 and 200 numbers of topics. Figure 1, 2, 3 and 4 shows that the CH-index values of LDA and LSA are lower than FLSA for the Genia dataset. The FKTM CH-index values are higher than FLSA for the Genia dataset, and MKFTM CH-index values are higher than FKTM for the Genia dataset. Therefore, the clustering performance of MKFTM is highest than other topic models like FKTM, FLSA, LDA, and LSA for the Genia dataset. Figures 1, 2, 3, and 4 indicate that the CH-index values of LDA and LSA are lower than those of FLSA for the Genia dataset. For the Genia dataset, the FKTM CH-index values are greater than the FLSA. For the Genia dataset, MKFTM CH-index values are greater than FKTM. As a result, MKFTM outperforms other topic models for the Genia dataset, like FKTM, FLSA, LDA, and LSA in terms of clustering performance. Figures 5, 6, 7, and 8 show that the CH-index values of LDA and LSA are lower than FLSA for the Biotext dataset. For the Biotext dataset, the FKTM CH-index values are greater than the FLSA. MKFTM CH-index values are greater than FKTM for the Biotext dataset. As a result, for the Biotext dataset, MKFTM outperforms other topic models like FKTM, FLSA, LDA, and LSA for clustering. Therefore, MKFTM achieved better clustering performance for Genia and Biotext datasets.

Table 4 shows that log-likelihood for the WSJ dataset with 50, 100, 150, and 200 topics. The log-likelihood results of MKFTM are better than the FKTM, FLSA, LDA, and LSA with different topics. Therefore, MKFTM also solves the redundancy issues and achieves better performance for redundant corpora than FKTM, FLSA, LDA and LSA.

The execution time performance for the health news tweets dataset is shown in Fig. 9. The execution time performance is measured with 50, 100, 150, 200, 250, 300, and 350 numbers of topics. The execution time of LDA and LSA is increased as the number of topics increases, but the execution time of MKFTM is stable.

## Conclusion

Biomedical text is on the rise these days, while evaluating these documents is extremely important to discovering valuable sources of information. Biomedical databases like PubMed provide valuable services to scientific communities. To reveal the hidden theme structures from biomedical text document topic modeling is a famous technique. These text documents used structured to search, index, and summarize. In advanced machine learning the fuzzy methods are mostly utilized in medical imaging. The existing topic modeling method is based on linear and statistical distribution. This paper presented a new multiple kernel fuzzy topic modeling (MKFTM) approach for biomedical text documents. We also proposed a new fusion probabilistic inverse document frequency. MKFTM improves the negative consequences of redundancy words for biomedical text documents and perform better than LDA and RedLDA. MKFTM also remove the sparsity problem in biomedical text documents. Experimental results indicate that MKFTM performs better in biomedical documents' classification and clustering tasks than the state-of-the-art topic models LDA, LSA, FLSA and FKTM. MKFTM is a new approach to topic modeling, which has the flexibility to work with a variety of clustering and scaling techniques. Furthermore, the MKFTM method uses discrete and continuous data to extract topics from biomedical documents. The six datasets quantitative evaluation describes that MKFTM performs better than progressive baselines with significant improvements.

## Data Availability

All datasets generated or analysed during this study are publicly available and discussed in materials and method section.

## Footnotes

1 http://muchmore.dfki.de/resources1.htm

2 http://disi.unitn.it/moschitti/corpora/ohsumed-first-20000-docs.tar.gz.

3 https://archive.ics.uci.edu/ml/datasets/Health+News+in+Twitter
